# Matching SDN and Legacy Networking Hardware for Energy Efficiency and Bounded Delay †

**DOI:** 10.3390/s18113915

**Published:** 2018-11-13

**Authors:** Pablo Fondo-Ferreiro, Miguel Rodríguez-Pérez, Manuel Fernández-Veiga, Sergio Herrería-Alonso

**Affiliations:** atlanTTic Research Center, University of Vigo, 36310 Vigo, Spain; miguel@det.uvigo.gal (M.R.-P.); mveiga@det.uvigo.es (M.F.-V.); sha@det.uvigo.es (S.H.-A.)

**Keywords:** energy-efficient Ethernet, QoS, SDN, real-time traffic, ONOS

## Abstract

Both economic and environmental costs are driving much research in the area of the energy efficiency of networking equipment. This research has produced a great amount of proposals. However, the majority of them remain unimplemented due to the lack of flexibility of current hardware devices and a certain lack of enthusiasm from commercial vendors. At the same time, Software-Defined Networking (SDN) has allowed customers to control switching decisions with a flexibility and precision previously unheard of. This paper explores the potential convergence between the two aforementioned trends and presents a promising power saving algorithm that can be implemented using standard SDN capabilities of current switches, reducing operation costs on both data centers and wired access networks. In particular, we focus on minimizing the energy consumption in bundles of energy-efficient Ethernet links leveraging SDN. For this, we build on an existing theoretical algorithm and adapt it for implementing with an SDN solution. We study several approaches and compare the resulting algorithms not only according to their energy efficiency, but also taking into account additional QoS metrics. The results show that the resulting algorithm is able to closely match the theoretical results, even when taking into account the requirements of delay-sensitive traffic.

## 1. Introduction

Nowadays, public concern about energy consumption of networking equipment is increasing due to not only environmental reasons, but also economic ones. Inside data centers, the network consumes up to 20% of its total power [1]. If we focus on wireless networks, the reduction of energy consumption is also a Key Performance Indicator (KPI) according to the 5G Infrastructure Public Private Partnership (5G-PPP) [2]. As a result, a wide range of solutions has been proposed to reduce the energy consumption of networking equipment. However, many of these new techniques remain unimplemented due to the lack of flexibility in current networks. For instance, some of the recent proposals in the literature require changes to basic protocols, whereas others require changes to the networking equipment [3,4,5,6].

In parallel, the Software-Defined Networking (SDN) paradigm is being embraced by the networking community. The SDN paradigm moves the forwarding logic from the devices themselves to the SDN applications, which run on top of the logically-centralized controller. In these networks, switches are just pure forwarding fabrics instructed by the SDN controller, and the network policies are programmed through software applications, which run on top of the SDN controller. The flexibility that this paradigm introduces has led to its extensive adoption in data centers. SDN is also deemed as a key enabler technology for 5G networks [7]. The adoption of SDN in these networks is seen as an opportunity to improve the energy efficiency of the communications infrastructure, overcoming the limitations of current networks by virtue of its programmability and flexibility.

Energy-Efficient Ethernet (EEE) [8,9] is the standard for saving energy in Ethernet interfaces. Despite the large savings in energy consumption achievable with EEE over single Ethernet links, the overall consumption in Ethernet link aggregates is not proportional to the offered load and depends largely on the actual traffic share among the links. In this paper, we adapt efficiently an analytical solution to the problem of minimizing energy in bundles of EEE links by leveraging the operational principles of SDN networks. Therefore, combining analysis with SDN capabilities, legacy switches equipped with EEE line cards can run energy-aware traffic distribution algorithms even if the vendors do not build support for them in the hardware/firmware. Specifically, we design, build and analyze three energy-efficient SDN-compatible flow allocation algorithms from the point of view of energy consumption, packet loss rate and transmission latency, both through simulations and also with an implementation on top of the Open Network Operating System (ONOS) SDN controller. Since the energy saving nature of the algorithms can make latency increase, we also consider traffic with different QoS requirements. Subsequently, two different solutions are proposed to handle the low-latency traffic while at the same time reducing the energy consumption. Our solutions are validated both through extensive simulation experiments and by implementing the algorithms in ONOS.

This paper extends our previous work presented in [10,11] by discussing further the related work, describing the solutions in detail, studying a new mechanism for estimating the rate of each flow and providing a thorough analysis of the algorithms. The rest of the paper is organized as follows. Section 2 introduces the related work. We describe our proposal for minimizing energy consumption in Section 3. Section 4 shows the QoS-aware algorithms. Results are discussed in Section 5. Finally, we draw some conclusions in Section 6.

## 2. Related Work

The advantages offered by SDN networks for advanced traffic management have been the subject of study of prior works that helped to understand the best way to use SDN for spending less energy. In [12], the authors carried out a survey on energy efficiency, identifying which components involved in the SDN networks can be configured in a dynamic way in order to reduce energy usage. Most of the solutions analyzed rely on re-routing the flows in the network so that the number of active switches is minimized. Thus, these devices can be put in a low-energy state or eventually turned off, reducing the power consumption of the network. These proposals are termed traffic-aware, since they need to know the traffic load that is currently passing through the network. The use of the centralized view of the topology provided by the SDN controller is a key assumption in this approach.

GreenSDN [13] is an emulation environment built using Mininet and POX, which is a Python-based SDN controller. The authors summarized the difficulties they found implementing an SDN environment with green capabilities. They presented an integration of three energy-saving protocols operating at different layers: adaptive link rate operating at the chip level, synchronized coalescing working at the node level and the sustainability-oriented network management system, which considers the whole topology to maintain the balance between QoS and energy savings. Particularly relevant to this paper is the mechanism proposed at the node level, which also exploits the Low Power Idle (LPI) state defined in the IEEE 802.3az standard. Nevertheless, GreenSDN does not consider setting individual ports in low power idle mode when the traffic traverses an aggregate between switches.

The energy-efficient flow routing problem is formulated in [14] as an optimization problem and solved with a heuristic—named the Energy Monitoring and Management Application (EMMA)—which aims to concentrate the traffic on the smallest possible set of nodes in the whole topology, so that the number of idle switches is maximized. EMMA is implemented as an ONOS application and evaluated in a network emulated through Mininet. The solution requires that the flows have previously declared their demanded rates. Thereby, when a new flow arises, EMMA tries to allocate all the active flows in the subset of the network topology that is currently active. If the active topology cannot support the flows, a new allocation for the whole network topology is computed. Analogously, when a flow is removed, EMMA attempts to re-route existing flows so that energy consumption of the network diminishes.

ElasticTree [1] and ECODANE [15] present solutions focused on data center networks, exploiting the redundancy in their internal switching networks and the variability in the workload that the data center must support over time. ElasticTree is a heuristic algorithm that adjusts the set of active devices to gauge the changes in the traffic load. The heuristic was validated over a testbed composed of production OpenFlow switches, using real traffic traces from an e-commerce website, saving up to 50% of energy. In ECODANE, an emulation framework, is built around Mininet and NOX, composed of five modules: the optimizer, which is in charge of determining the minimum subset of the topology that needs to be active, the power control module that manages the power states of the switches, the forwarding module that manages the flow rules installed in the switches to forward the traffic, the traffic generator, which generates the traffic to perform simulations, and the data center network itself. Their results obtained between a 10% and 35% energy reduction, depending on the source and destination of the traffic. However, none of these two solutions considers the characteristics of EEE links to reduce their energy consumption or explore the particular case of link aggregates between switches.

There is a clear line of work dedicated to exploring the interactions between resource activation/deactivation, routing decisions and energy savings. Early works focused on powering down redundant resources, e.g., switches and links, to reduce energy consumption during periods with low load. Some examples of these early works are [3,16,17,18], where the authors studied the energy savings obtained by link aggregation in metropolitan optical networks. All of these proposed different formulations to obtain the proper set of nodes to keep active, but only under the assumption of on-off power profiles in the devices. There is also research considering other more advanced link power profiles. For instance, transmission links with super-linear cost functions were studied in [19] with the goal of calculating the maximum power savings. New insights were provided in [20], after it was demonstrated that traffic consolidation can increase energy consumption for certain power profiles. The main problem with all these proposals was their high complexity, since the energy minimization problem is NP-complete [5]. Accordingly, a number of proposals in the literature resorted to heuristics for solving the problems. For instance, reference [21] took advantage of genetic algorithms to get close to the optimum, and [22] provided a heuristic based on particle swarm optimization. In general, these works overlook the problems derived from the practical implementation of the algorithmic results. Finally, only [23] reformulated the energy-saving problem considering an SDN-capable network and extended the problem to consider the usage of network function virtualization, so that computing tasks can also be moved across the network to enable greater energy savings.

A common feature of the prior works is that the underlying algorithms operate in relatively long time frames, and so, their response can be slow. Moreover, they address a network flow allocation problem, globally. In contrast, we focus on flow allocation in a single-link aggregate between two switches (see [6], which is the theoretical basis of this paper), and our techniques work in much shorter timescales, in the order of a single frame transmission. It was found therein that the optimum minimum-energy allocation of flows into a bundle of EEE links turns out to follow a water filling policy for typical consumption profiles, as explained in [6]. That is, a new port will only be used to transmit a packet if the packet cannot be transmitted by any of the ports already being used, since they are operating at its full capacity. This result holds for the main classes of mechanisms used to manage the power state of the IEEE 802.3az ports, i.e., frame transmission and burst transmission modes [24]. In addition, reference [6] presented an algorithm to achieve these results, which operates on a per-packet basis, deciding the port that will be used for each packet based on the occupation of the ports. Following a naive water filling algorithm and only diverting traffic to a new idle port when the previous ones are completely used at their full capacity will lead to an unbounded delay. Hence, a simple modification is proposed to maintain the average delay bounded to a target value, by using the average delay of the already queued packets to determine the output port.

## 3. SDN Application Design

In this section, we address the energy-efficient allocation of traffic flowing between two switches through an aggregate of EEE links, from the point of view of a software-defined network.

### 3.1. Background and Problem Statement

**Figure 1:**
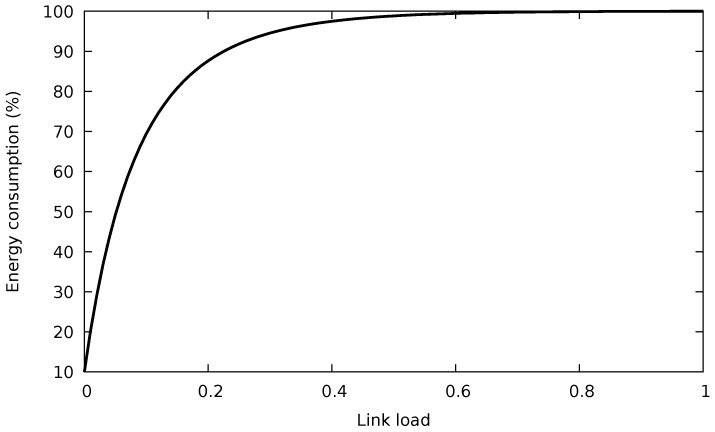
Consumption of a 10 Gb/s IEEE 802.3az port using frame transmission. © 2018 IEEE. Reprinted, with permission, from [10].

We will consider a link aggregate composed of *L* IEEE 802.3az links, of identical capacity *C*. The traffic traversing that bundle is represented by *F* flows. Let $x_{i}\in \left[0,C\right)$ denote the estimated rate of flow *i*, and let $p_{i}\in \left\{1,\ldots ,L\right\}$ be the port where that flow is allocated. According to [25], the normalized individual energy consumption of a single EEE interface for any uncorrelated incoming traffic distribution is:

(1) $$E\left(\rho \right)=1-\left(1-\sigma_{\mathrm{off}}\right)\left(1-\rho \right)\frac{T_{\mathrm{off}}\left(\rho \right)}{T_{\mathrm{off}}\left(\rho \right)+T_{S}+T_{W}},$$

where ρ is the traffic load, $\sigma_{\mathrm{off}}$ is the relative energy consumption of the EEE idle mode and $T_{S}$ and $T_{W}$ are the time needed to enter and exit the LPI mode, respectively. They are constant parameters dependent on the physical interface characteristics. Finally, $T_{\mathrm{off}}\left(\;\cdot \;\right)$ is the average length of the idle periods, which depends on both the algorithm governing the idle mode and the actual traffic load. Let $\vec{\rho }=\left[\rho_{1},\ldots ,\rho_{L}\right]$ be the load allocation to the links forming the bundle. Then,

(2) $$E_{B}\left(\vec{\rho }\right)=\frac{1}{L}\sum_{i=1}^{L}E\left(\rho_{i}\right)$$

is the normalized energy consumption of the whole bundle for a given load allocation. In [6], it was proven that, for any arbitrary algorithm governing the idle mode, $E_{B}\left(\;\cdot \;\right)$ is minimized iff:

(3) $$x_{i}=\min\left\{C,X-\sum_{j<i}x_{j}\right\},\;i=1,\cdots ,L,$$

where *X* denotes the total traffic allocation and $x_{i}$ is the traffic allocation to the *i*-th port, i.e., $\rho_{i}=x_{i}/C$. That is, the minimum energy consumption is obtained when a water filling algorithm is used to assign the traffic among the links in the bundle. This is because the typical energy consumption profile of a single Ethernet link is a concave function of the traffic load (see Figure 1). In addition to the theoretical solution, a practical algorithm is also provided by [6], but assuming that the switches operate on a packet-by-packet basis. For two reasons, this algorithm cannot be directly implemented as an SDN application:

- The ideal algorithm considers that the switch individually decides for each packet which port will be used to forward it, based on the instantaneous occupation of the ports, a packet-level operation. SDN does not allow forwarding each packet individually, since its data plane works at the flow level, applying the same actions to the packets of a flow once a matching rule is found in its flow table (i.e., forwarding the packets to the same port). In addition to the action prescribed by the flow rule, the counters associated with the port are updated.
- The current queue occupation of each port is used to determine the forwarding port. Unfortunately, this state variable is not usually provided by SDN switches (e.g., it is not considered in OpenFlow).

Throughout the rest of this section, we will present the main architecture of the SDN application and the new flow allocation algorithms that realize the solution presented in [6].

### 3.2. Designing the SDN Application

We devised a reactive forwarding behavior. That is, a low-priority rule will be installed in the switches to send the packets to the SDN controller, so the packets that do not match any flow rule with higher priority will be sent to the SDN controller, which has a centralized view of the topology and will act in response. The controller will transfer this packet to our application, which runs on top of the controller. Next, the application will determine which port the packet should be forwarded to and install a medium-priority flow rule in the switch. Future packets classified in this same flow will be directly forwarded by the switch at the line rate.

The medium-priority flows installed are defined by the destination MAC address of the packets and also the first eight bits of the destination IP address. Eight bits attain a good trade-off for the granularity of flows: enough to spread the traffic among the ports of the bundle and to keep the flow tables of the switches small to avoid performance degradation.

Since the controller maintains a full view of the network topology, it can compute shortest paths to the packet destination. For unknown destinations, the controller floods the packet out of all ports except the input port and using only one port of each bundle, without installing a flow rule for this packet yet. Therefore, when a packet is received, the application determines the next hop switch to which the packet should be forwarded. When the next hop is behind a bundle of links, our application selects at random a port of the bundle to forward the packet and installs the flow in the switch accordingly. The random selection is performed since the application does not have prior information about the transmission rate of this new flow. Allocating the flow to the highest loaded port would cause excessive losses if the flow demands a high rate. On the contrary, using an idle port would activate its hardware, drastically increasing the energy consumption if the flow demands a rate that can be handled by ports already active. In any case, it is important to note that this is just an initial transient when a new flow appears on the network.

**Figure 2:**
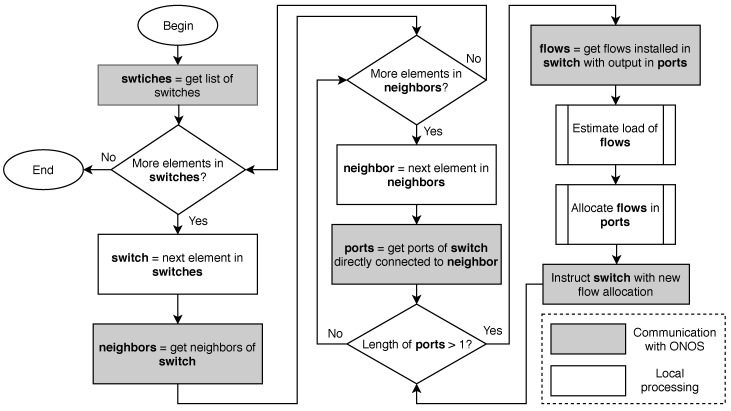
High level application logic.

The above description is the part of the SDN application that manages the packet forwarding in the transport infrastructure. It uses a customized flow definition, but spreads flows at random in the bundles between switches since there exists no a priori information about the flow rates, thus not performing any energy-aware optimization. Now, we proceed to describe how to optimize the energy consumption in the bundles. Figure 2 displays the flow diagram of the control application. The application performs the following tasks, some of them periodically:

1. Retrieve the list of switches.
2. For each switch, identify the neighbors of the switch (i.e., the switches that a link to it).
3. For each neighbor, retrieve the ports in the switch that are connected with the neighbor.
4. If there is more than one port (i.e., there is a bundle between the two switches), retrieve the flows installed in the switch that forward packets to a port of this bundle.
5. Predict the rate of each flow; that is to say, the amount of traffic that the flow will transmit in the next interval.
6. Compute a new allocation for these flows to the ports of the bundle in a way that energy consumption is minimized.
7. Instruct the switch to modify the flow rules that have changed their allocation.

Two of these deserve further discussion: rate prediction and flow allocation.

### 3.3. Flow Rate Prediction

The rate demanded by a flow in the following measurement interval is estimated leveraging the counters associated with the flow rules. These counters include the number of packets and bytes that have matched with this flow along with the duration of the flow (i.e., the time that the flow has been active). Thus, the bytes transmitted in the current measurement period are the difference between two counter queries by the controller. The estimated rate is simply the number of transmitted bytes divided by the sampling period. If the flow was not present in the previous interval, we use the value of the duration of the flow instead of the sampling period, for an accurate measure. For the prediction of the flow rate in the next measurement period, we tested two simple estimators:

1. The measured value in the previous interval.
2. An exponentially-weighted moving average (EWMA) with the measured rates of the flow in past intervals. The estimated rate is:
  (4) $$R_{n}=\left\{\begin{array}{cc} M_{n}, & n=0\\ \alpha \;\cdot \;M_{n}+\left(1-\alpha \right)\;\cdot \;R_{n-1}, & n>0 \end{array}\right.$$

where $R_{n}$ is the value of the EWMA at time *n* (thus, the rate estimated for the interval n+1), $M_{n}$ is the measured rate in the interval *n* and the constant α∈(0,1] is a parameter that tunes the relevance of the samples as time goes by.

**Figure 3:**
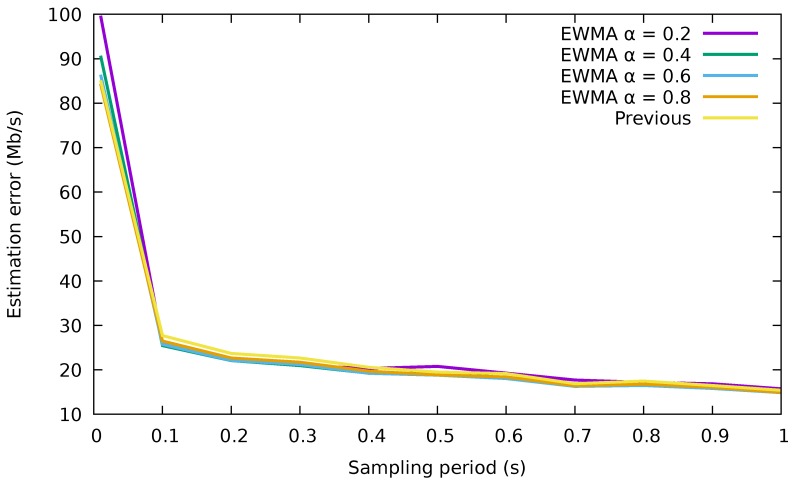
Average error in the estimation of the flow rate for different periods. EWMA, exponentially-weighted moving average.

We have analyzed the quality of the estimation with the two methods, calculating the error in the estimation as the absolute value of the difference between the estimated value and the real value, for each flow in each interval. Just for a reference, the results for a 32.5 Gb/s trace are shown in Figure 3 (Although we have not included them for space reasons, results for several other traffic traces have been produced with similar results. The traffic traces are the same as those used in Section 5, where their exact characteristics are described).

Clearly, using directly the measurements performs very similar to the usage of the EWMA for the different values of α and the sampling periods studied. We can also notice large errors in the estimated rate for sampling periods lower than 0.1 s. This is due to the high variability of the rates for such a small time window. Therefore, we will directly use the rate of each flow in the previous interval to forecast its rate in the next interval, since it is a simpler method than the EWMA one and provides almost the same accuracy.

### 3.4. Flow Allocation Algorithm

In this section, we describe three new flow allocation algorithms implemented to reduce the energy consumption. The purpose of these algorithms is to select the flows according to their traffic rate and assign them to the available links according to the optimum flow allocation algorithm [6]. The input of this algorithm will be the set of flows to be allocated along with their estimated rates, and also the set of ports that make up the aggregate.

#### 3.4.1. Greedy Algorithm

GA attempts to fill the ports to the maximum of their capacity, only allocating a flow in an empty port if it does not fit in any of the already active ones. Therefore, GA attempts to use only the minimum number of ports, filling them up to their nominal capacity and leaving the maximum number of ports empty. The latter problem is actually a combinatorial NP-hard problem (bin packing), so we propose using the first-fit decreasing (FFD) [26] algorithm as a heuristic suboptimal solution. Note that finding the optimum one would require evaluating ${\left|\mathrm{ports}\right|}^{\left|\mathrm{flows}\right|}$ combinations and is not scalable. Instead of this, our GA algorithm will consider the flows in a decreasing order based on their estimated rates and will allocate them to the highest loaded port. Note that although similar to a water filling approach, GA does not operate at the packet level, but at the flow level.

**Figure 4:**
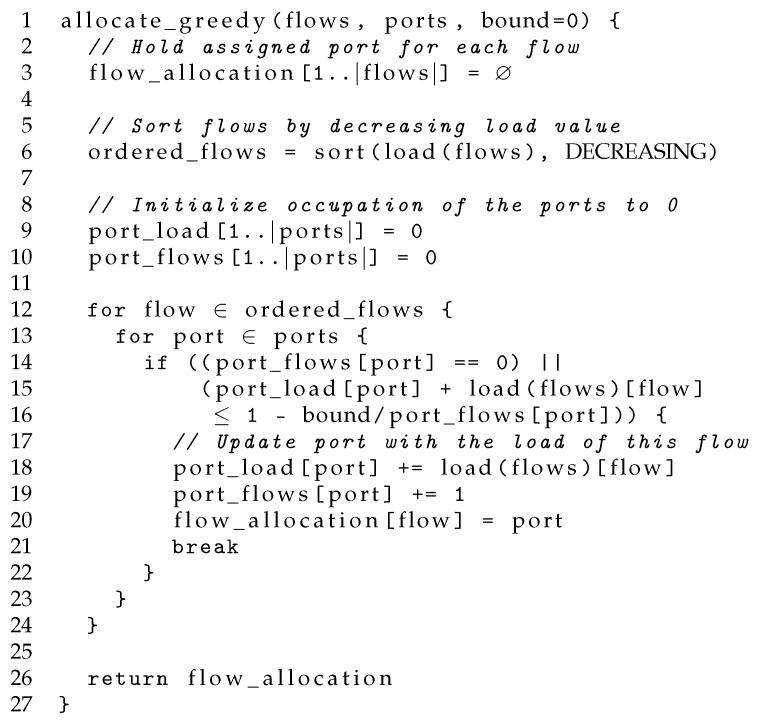
Pseudocode for the greedy algorithms. Note that the greedy algorithm (GA) is identical to bounded GA (BGA) when setting the variable bound to zero. © 2018 IEEE. Reprinted, with permission, from [10].

The pseudocode of the GA algorithm is shown in Figure 4, where the bound is always set to zero. Firstly, the flows are sorted decreasingly on their estimated rate. Next, the flows are allocated in a sequential order so that the port occupation is maximized: the ports are evaluated in a predefined order (e.g., by port identifier), and the flow is allocated to the first port it fits. We consider that a flow fits in a port when the sum of all the flow rates assigned to the port is less than the capacity of the port. This algorithm is akin to the classical FFD heuristic solution of the bin packing problem [26]. We expect GA to yield low values of energy consumption. Nevertheless, since it pushes to use the full capacity of the ports, packet delay can grow quickly, as pointed out in [6]. Furthermore, since rate estimations are noisy, the estimation errors could lead to a non-negligible level of packet losses for almost any buffer size.

#### 3.4.2. Bounded Greedy Algorithm

BGA is a straightforward modification of GA, in that it attempts to bound the packet delay and also to reduce the packet losses in GA. One reason for the losses is that using the ports very close to their capacity leads easily to buffer overload if the rate is not accurately estimated. Thus, BGA avoids using links close to their capacity just by setting a threshold in the maximum allowable load allocated on a port. Specifically, we limit the fraction of the port capacity that can be used to an increasing function in the number of flows already allocated to each port *p*, with the following function:

(5) $$\rho_{\max}^{p}=1-B/F_{p},$$

where $F_{p}$ is the number of flows already allocated to the port and *B* denotes the fraction of space that cannot be used in a port when there is only one flow allocated to it. The idea is that rate prediction errors between the different flows should compensate the global estimation error, and thus, the higher the number of flows, the higher the link occupation that is safe to attain. For the rest, the algorithm operates in the same way as GA. The pseudocode is also shown in Figure 4, where *bound* is the fraction of space that cannot be used in a port when there is only one flow allocated to it, i.e., *bound* =B.

#### 3.4.3. Conservative Algorithm

Despite the effort of the BGA to mitigate packet losses and control the delay of the packets, the results might not be acceptable yet, as we will later show. Hence, we designed a better algorithm that does not only minimize energy consumption, but also reduces packet losses. The idea behind this algorithm is to first compute the minimum number of ports necessary for the next interval. This value is lower bounded by:

(6) $$L_{\mathrm{used}}=\left\lceil \frac{\sum_{i=1}^{F}x_{i}}{C}\right\rceil .$$

Then, the flows are distributed among the $L_{\mathrm{used}}$ ports in a way that tries to spread the load evenly. Although this minimizes the individual occupation of each link, it does not follow a water filling approach. However, as we will show later, this does not really degrade energy consumption. The reason is that the individual energy consumption rises very quickly with the occupation of the link, as Figure 1 illustrates.

**Figure 5:**
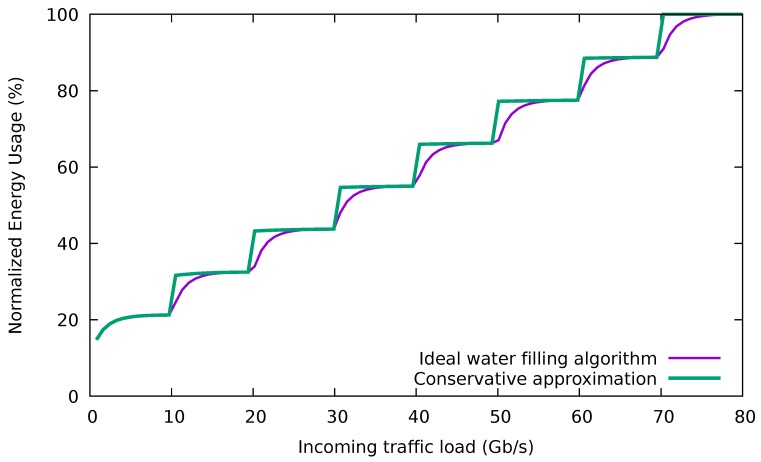
Comparison between the normalized power profiles of an eight-link bundle when using the water filling and the conservative algorithm.

As a consequence, once a port reaches an occupation higher than 20%, it makes little difference for the energy consumption the actual traffic load assigned to it. Thus, the conservative algorithm (CA) prefers to have its used ports with a balanced traffic occupation avoiding the need for using the ports at full capacity in many situations, like frequently happens with the GA and BGA. Figure 5 shows normalized power profiles of an eight-link bundle with the ideal water filling algorithm and the power profile expected for the conservative algorithm.

For further reduction of the likelihood of packet losses, rather than using the total estimated load to compute the number of ports, we add to this value a safety margin, *M*, which we empirically set to 20%. The number of ports to be used is calculated therefore as:

(7) $$L_{\mathrm{used}}=\left\lceil \frac{\sum_{i=1}^{N}x_{i}}{C}+M\right\rceil .$$

**Figure 6:**
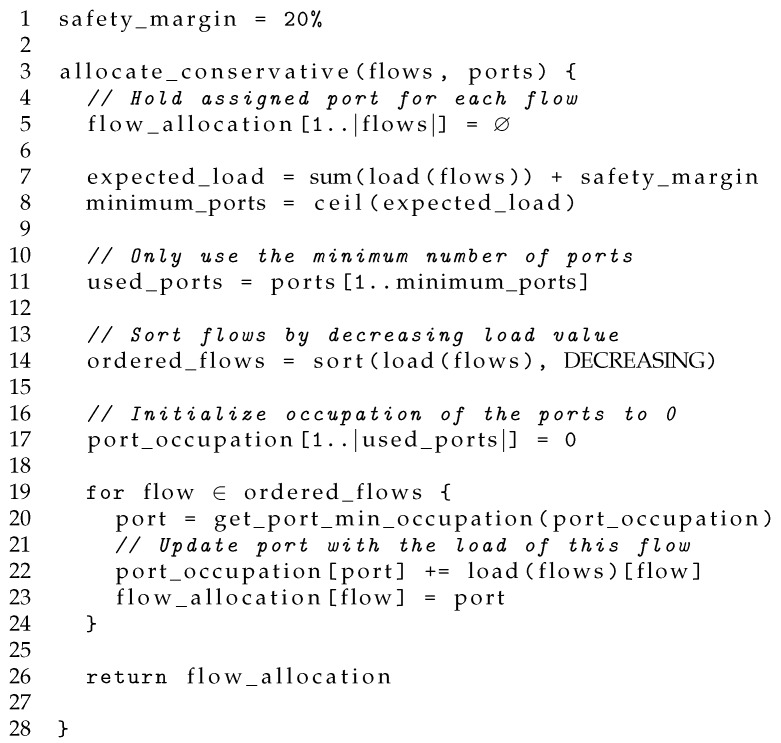
Pseudocode for the conservative algorithm. © 2018 IEEE. Reprinted, with permission, from [10].

After determining the $L_{\mathrm{used}}$, the CA proceeds with the allocation of the flows attempting to achieve a balanced distribution of the flows to ports, both in terms of rate and number of flows. To accomplish this, the algorithm performs a minimization of the occupation of the ports. The pseudocode is shown in Figure 6, which first sorts the flows in decreasing order of their estimated rate and then sequentially allocates the flows to the port with the lowest occupation among those that will be used in the interval. Note that this algorithm is also capable of maintaining some ports idle, reducing energy consumption.

## 4. Energy-Efficient Algorithms with Bounded Delay

In this section, we consider flows with different quality of service (QoS) requirements in terms of latency. We will introduce two modifications to the energy-efficient algorithms described in Section 3 in order to consider the demands of low-latency flows. The specific mechanism used to identify low-latency flows is not relevant for this work since the method actually employed does not affect the algorithm at all. Thus, we will assume that the low-latency flows are tagged with a well-known differentiated services code point (DSCP), which is carried in the IP header. As a result, we will work with two types of flows: low-latency flows and best-effort flows.

These modifications are generic enough to be applied to any of the different energy-efficient algorithms presented above. However, since CA outperforms the other two algorithms in packet losses and delay with a minimum penalty in energy savings, as will be later shown in Section 5.1, during the rest of this paper, we will only use CA as the energy-efficient algorithm, for the sake of simplicity.

### 4.1. Spare Port Algorithm

**Figure 7:**
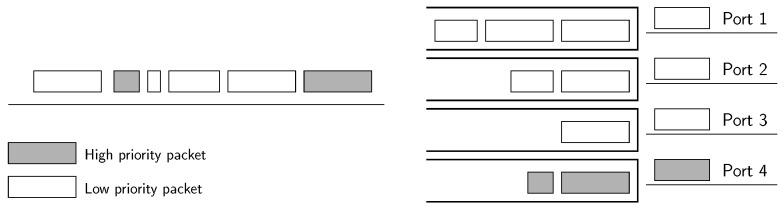
Conceptual diagram of the spare port algorithm in a four-link bundle.

The spare port algorithm (SPA), shown in Figure 7, exploits the fact that, when the traffic load is moderate, the energy-efficient algorithms concentrate the traffic and leave some spare ports. Therefore, this new algorithm trades an increase of energy consumption in unused ports so as to provide expedited service to low-latency flows. SPA works in two phases, first allocating best-effort flows and then the low-latency ones.

1. In the first phase, the energy-saving algorithm is directly applied without modifications, but only to the best-effort flows.
2. In the second phase, the remaining low-latency flows are assigned to the least occupied port among those in the bundle.

SPA can perform well under the assumptions that low-latency traffic represents a small fraction of both the total traffic and the spare port capacity and that best-effort flows do not exhaust the capacity of the bundle. Then, in the next step, one of the unused ports will be used to forward low-latency traffic, without increasing the delay of the best-effort traffic. However, we need to discuss the limitations of this algorithm when some of the assumptions do not hold:

1. If the traffic demand is so high such that all the ports in the bundle must be dedicated to best-effort flows, low-latency traffic will not be forwarded through a single port. As a result, both low-latency and best-effort traffic will be treated in the same way, without meeting the needs of premium traffic.
2. If the amount of low-latency traffic is significant, the energy consumption of the spare port can drastically increase because of the energy profile of an EEE link, which rises very quickly with the port occupation (cf. Figure 1).

### 4.2. Two Queues Algorithm

The spare port algorithm may increase the energy consumption if the amount of delay-sensitive traffic is significant. More importantly, SPA will not be able to satisfy the demands of flows with low-latency requirements when there is a high load due to best-effort traffic. To solve this, we leverage the ability of most SDN switches to have multiple queues attached to a physical port. These queues can be defined with different priorities. In fact, this is the standard way of providing QoS in SDN devices as stated in the OpenFlow (OF) specification [27]. Although this capability is not required, it is provided by most of the devices, such as Open vSwitch, which is presumably the most widely-used OF-enabled switch.

**Figure 8:**
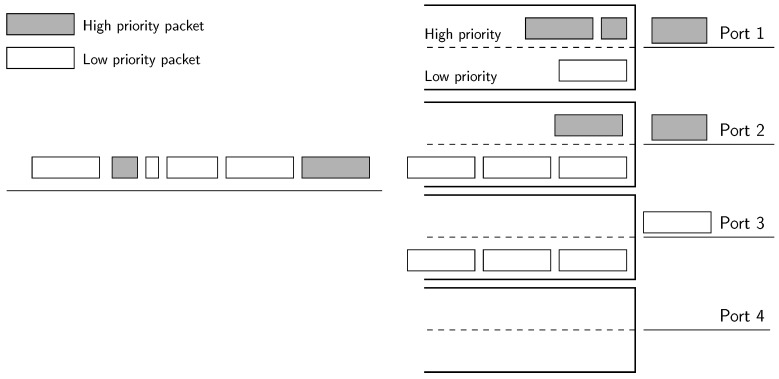
Conceptual diagram of the two queues algorithm in a four-link bundle.

In the two queues algorithm (TQA), we define two queues with different priorities inside each physical port of the switches, as shown in Figure 8: the queue with the highest priority will only serve low-latency traffic, and the other will forward best-effort traffic. The algorithm operates in two phases, determining first the port and then the queue inside each port:

1. The first phase consists of directly applying the unmodified energy-efficient algorithm described in Section 3 to the whole set of flows, both including low-latency and best-effort, treated equally. The whole set of flows is allocated in a few ports.
2. The second phase sets the queue inside the assigned port for every flow. Low-latency flows are assigned to the high-priority queue of the ports, whilst best-effort flows are assigned to the low-priority queue.

Clearly, the allocation of flows to ports is actually given by the energy-efficient algorithm, but thanks to the introduction of multiple first-in first-out (FIFO) queues inside the port, we prioritize flows with stringent QoS requirements in terms of latency, thus providing an expedited service. The decision of the next packet to be served by a port is straightforward: each time the port ends the transmission of a packet, it will pick the next packet to be transmitted from the non-empty queue with the highest priority. In other words, delay-sensitive packets have non-preemptive priority over the rest.

Using two queues per port overcomes the limitations of SPA. Firstly, the service received by the high-priority traffic is independent of the amount of best-effort traffic load. Secondly, the expedited service does not lead to increased energy consumption, since the joint allocation phase still uses the energy-efficient algorithm. Thus, the energy consumption will be equal to the original energy-efficient algorithm described in Section 3. The main drawback is that using two queues increases the delay of best-effort traffic, and this becomes more noticeable as the share of traffic demanding expedited service is higher. However, since the use of the priority queues only implies a reordering of the packets in a port, the average delay of all the packets will not change. Thus, the maximum delay of best-effort packets is still bounded.

## 5. Results

Throughout this section, we analyze the performance of the proposed algorithms to then assess the correct behavior of the proper ONOS application.

**Figure 9:**
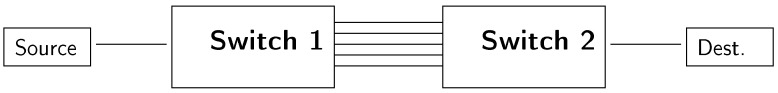
Basic experimental topology.

The algorithms have been analyzed in a scenario composed of bundle of five 10 Gb/s copper based Ethernet links (10 GBASE-T) aggregated between two switches with two hosts attached to them, one of them serving as the source for the traffic, and the other one acting as the traffic sink. Figure 9 shows a diagram of the setup.

The experiments have been carried out with an in-house developed simulator available for download [28]. The simulator shares most of the code with the ONOS implementation, thus reducing the developing time and helping in the validation. As for the traffic itself, we have employed real traffic traces from the public CAIDA (Center for Applied Internet Data Analysis) dataset [29]. As the original traces have been captured in 10 Gb/s Ethernet links, they have a relative low average throughput, so we have constructed new traffic traces, reducing the inter-arrival times by a constant factor, producing new 6.5, 13, 19.5, 26 and 32.5 Gb/s traces.

### 5.1. Flow Allocation Algorithms

**Figure 10:**
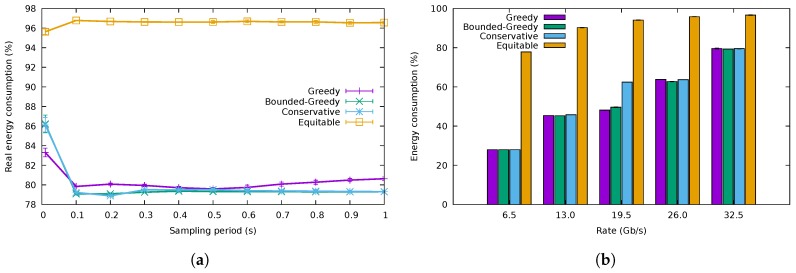
Energy consumption. (**a**) Energy consumption for different sampling periods, (**b**) Energy consumption for different traffic traces.

The first experiment evaluated the variation of the energy consumption with the duration of the sampling period of the algorithms for a rate of 32.5 Gb/s. Figure 10a shows the results of the three flow allocation algorithms for a buffer size of 10,000 packets. There was a fourth algorithm, named equitable, that distributed the flows uniformly among all the ports without regards to energy efficiency, serving as a baseline for comparison. The energy consumption attained by the three energy-saving algorithms was practically the same. Besides, we can also observe from Figure 10a that low sampling periods (e.g., lower than 0.1 s) presented higher consumption than those greater than 0.1 s. This probably corresponds to mispredictions of the flow characteristics, as already shown in Figure 3. Finally, note that the obtained energy consumption was very close to the optimum. According to (3), the best allocation was obtained when the rate allocation vector was $\vec{x}=\left[10\;Gb/s,10\;Gb/s,10\;Gb/s,2.5\;Gb/s,0\;Gb/s\right]$; in other words, this minimum consumption was achieved when the 32.5 Gb/s load was distributed in the bundle in the following way: three ports fully utilized carrying 10 Gb/s, one transmitting 2.5 Gb/s and the last one with no traffic. In that case, and for the usual EEE parameters ($\sigma_{\mathrm{off}}=0.1$, $T_{W}=4.48$ μs, $T_{S}=2.88$ μs for 10 Gb/s links and $T_{\mathrm{off}}\left(\lambda \right)=\lambda^{-1}e^{-\lambda T_{S}}$ for the frame transmission mode [25]), the bundle consumption was $\frac{1}{5}(1+1+1+0.83+0.1)=0.78$ using (2). This is just a little less than the 79% energy consumption obtained by CA.

The energy consumption for the different traffic traces is shown in Figure 10b for a sampling period of 0.5 s and a buffer size of 10,000 packets. We can observe that the energy consumption was almost identical for the three proposed algorithms and that it was considerably lower than that of the non-energy-efficient equitable algorithm. There was just a slight difference in the case of the 19.5 Gb/s, where CA consumed a bit more than the greedy algorithms. This was because its safety margin (*M*) made it use three ports, while the greedy algorithms would try to allocate the flows using just two ports. Nevertheless, the consumption attained by CA was indeed much lower than that of the equitable one.

**Figure 11:**
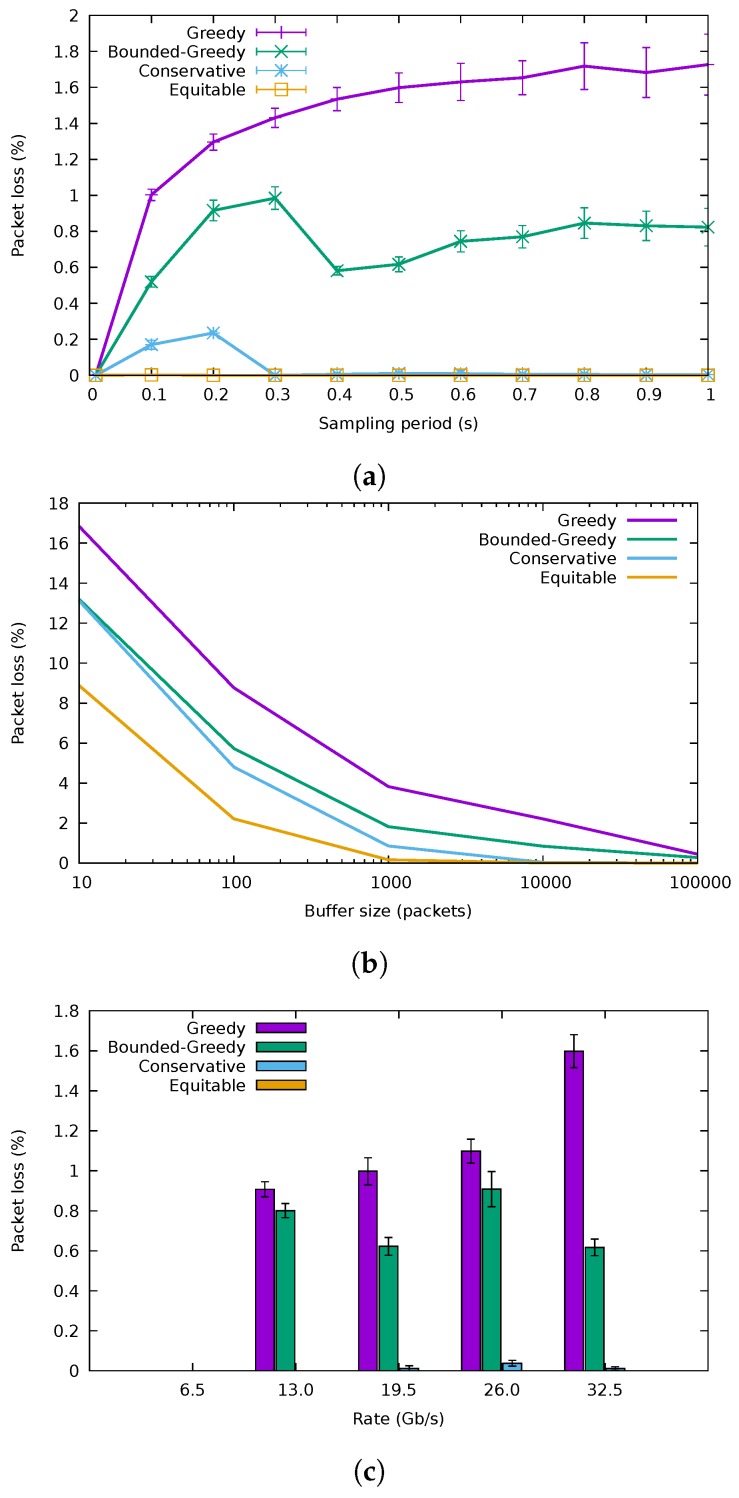
Packet loss rate. (**a**) Packet loss percentage variation with the duration of the sampling period. (**b**) Packet loss percentage variation with the buffer size. © 2018 IEEE. Reprinted, with permission, from [10]. (**c**) Packet loss for different traffic traces.

Figure 11a presents the variation of the packet loss rate with the sampling period for a 10,000-packet buffer size, while Figure 11b explores the packet losses introduced for different buffer sizes, using a sampling period of 0.5 s. GA was the one with the highest losses, followed by BGA, then CA and finally the equitable algorithm. These results confirm that the greedy algorithms can lead to high loss rates when the flow rates are underestimated. The conservative algorithm, however, was able to trade a small increment in energy consumption for an acceptable loss rate for buffer sizes from 1000 packets onward. Furthermore observe how for the highest sampling rates, packet losses diminished, as the algorithm adapted faster to rate variations; although, as seen before, energy usage also incremented.

The packet loss rates for the different traffic traces are shown in Figure 11c, where the sampling period is set to 0.5 s and the buffer size to 10,000 packets. As expected, GA and BGA were the ones having the highest losses in every case, with CA and equitable algorithms showing negligible losses. In the case of the 6.5 Gb/s trace, losses were not recorded, as expected.

**Figure 12:**
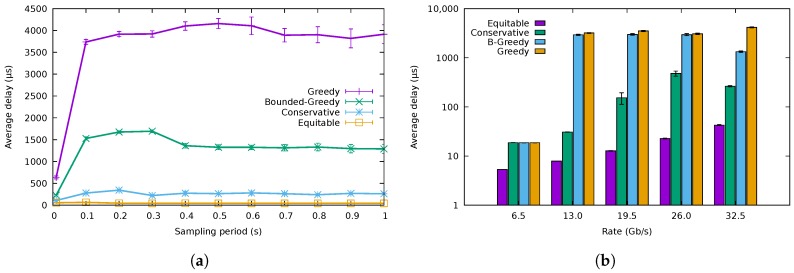
Packet delay. (**a**) Packet delay for different sampling periods, (**b**) Average packet delay for the different traffic traces.

The results for packet transmission delay are depicted in Figure 12. In particular, Figure 12a shows average packet delay variation versus sampling period for a 10,000-packet buffer size. The average delay for GA was about 4 ms, which is considerably higher than that of the other algorithms, whereas the delay for BGA was about 1.5 ms, which is still a high value. The delay for CA was, however, an order of magnitude lower, about 250 μs. For reference, the delay of the equitable algorithm sat around 50 μs, being, as expected, the lowest one. Figure 12b shows the average packet delay experienced by the different traffic traces with the different algorithms. For the 6.5 Gb/s trace, the three energy-saving algorithms behaved identically, using just one port for all the traffic. Furthermore, for CA, the delay of the packets using the 26 Gb/s trace was higher than that using the 32.5 Gb/s one, as, in the latter case, there was one more link in use, but with lower load. For the rest of the traces, the results were in accordance with those shown in Figure 12a.

### 5.2. QoS-Aware Algorithms

To test the performance of the two proposed QoS scheduling algorithms, we have created an additional traffic trace of 45.5 Gb/s reducing again the inter-arrival times of the original CAIDA trace. Additionally, we have added a source of low-latency traffic, consisting of a synthetic traffic trace made of relatively small packets (100 and 200 bytes) and deterministic inter-arrival times corresponding to the desired final average rate. We have used CA as the flow allocation algorithm using a sampling period of 0.5 s and a buffer size limited to 10,000 packets to provide negligible (below 0.05%) packet losses, as per the results of the previous section.

**Figure 13:**
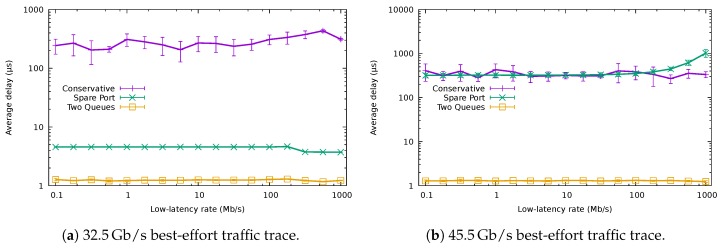
Average delay of the low-latency traffic.

Figure 13a shows the average delay of the packets with low-latency requirements using the QoS-aware algorithms and that obtained using the baseline conservative one. The results in the figure correspond to the best-effort traffic trace of 32.5Gb/s, while we varied the rate of the low-latency traffic (We have omitted the results using lower rates for the best-effort traffic for the sake of brevity, since the results are analogous). The unmodified CA yielded considerably worse results than the QoS-aware algorithms, producing a delay higher than 100 μs. The fluctuations for the different rates of the low-latency traffic come from the fact that the low-latency flows would be allocated to a different port in each case, being forced to compete with a different amount of normal traffic.

Both QoS-aware algorithms significantly reduced the average delay. The SPA delay stayed around 5 μs, while TQA added less than 2 μs for all the tested transmission rates. The main delay contribution for SPA was the time needed to wake up the interface (TW=4.48 μs), which would be usually idle at the arrival of a low-latency packet. This was not the case for TQA, as low-latency traffic shared the port with best-effort traffic.

Figure 13b shows the results when the system is already experiencing a very high load due to best-effort traffic ( 45.5Gb/s). Both SPA and the non-QoS-aware CA experimented with an average delay higher than 200 μs, fluctuating up to 1000 μs, depending on the actual low-latency rate. On the other hand, TQA maintained the latency lower than 2 μs. These results confirm that SPA was not capable of providing a low latency service in high load scenarios, since all the ports were already busy forwarding best-effort traffic.

**Figure 14:**
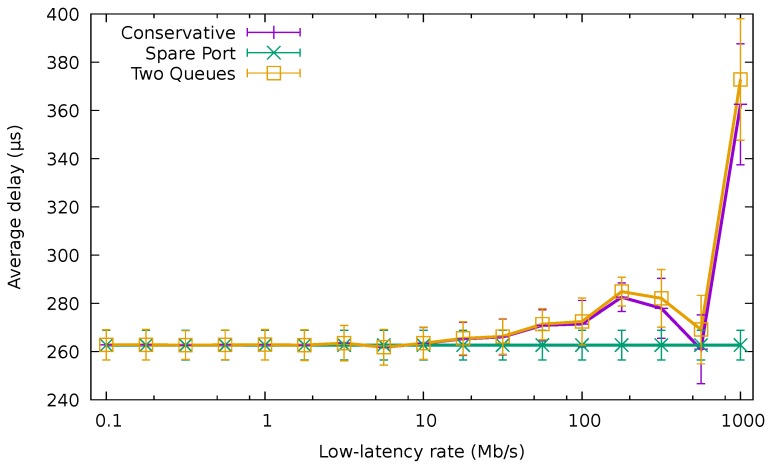
Average delay of the normal packets for the 32.5 Gb/s best-effort traffic trace.

Figure 14 compares the average delay of the best-effort packets using the QoS-aware algorithms with the average delay of these packets when using CA for the 32.5 Gb/s best-effort traffic trace and varying low-latency traffic. When the rate of low-latency packets was very low (e.g., lower than 100 Mb/s), the average delay of best-effort packets was identical for the two QoS algorithms and CA, being around 264 μs. Nevertheless, as this rate increased, the delay exhibited by CA and TQA rose. On the other hand, the delay of the SPA remained unaffected by the rate of the low-latency traffic, since it was being forwarded through a different port than the best-effort traffic.

**Figure 15:**
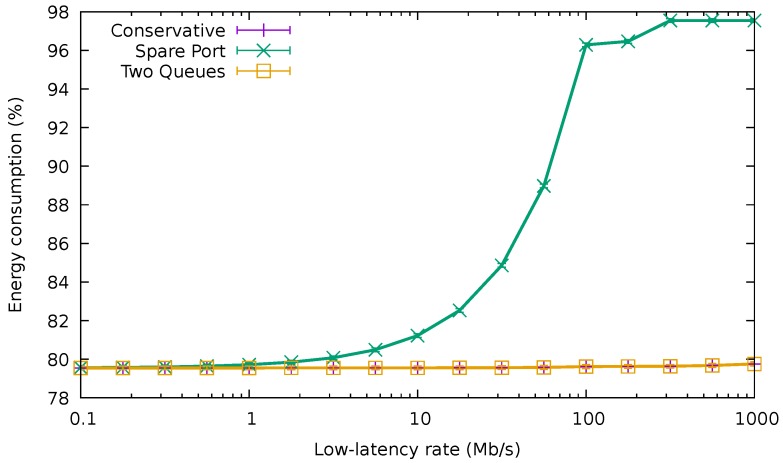
Normalized energy consumption for the 32.5 Gb/s best-effort traffic trace.

Finally, Figure 15 shows the average energy consumption of the bundle using the different QoS-aware algorithms and also CA in the same traffic conditions. Again, while the amount of high-priority traffic was negligible (i.e., lower than 10 Mb/s), the three algorithms drew the same amount of energy. As expected, TQA achieved exactly the same consumption as CA irrespective of the low-latency traffic rate. However, for values higher than 10 Mb/s, the energy usage increased rapidly for SPA, reaching nearly 100% for rates above 100 Mb/s. This confirms that energy consumption can rise quickly in SPA as soon as the amount of high-priority traffic forwarded in the spare port becomes significant.

### 5.3. ONOS Application Results

The previous sections have measured the efficiency of the proposed algorithms via a simulation study. We also tested the correctness and feasibility of the proposal with an actual implementation of the application. To this end, we implemented the proposed SDN application on top of the ONOS, emulating the experimental topology with Mininet in order to evaluate the proper operation of the application. The Open vSwitch switches employed by Mininet have an OpenFlow API accessible by ONOS, but it cannot reproduce exactly the EEE capabilities, so we measured the average occupation of each outgoing link as a proxy for the corresponding energy consumption.

We evaluated our application with the 32.5 Gb/s traffic trace used in the previous experiments (Results for the other traffic traces, namely the 6.5, 13, 19 and 26 Gb/s ones, have been omitted for the sake of brevity, but otherwise show consistent results).

We used tcpreplay to transmit it, but at a rate of just about 330 Mb/s, since the computer used for the experiments was not capable of transmitting this traffic trace at higher rates (We have used an Intel® Core™ i7-4710HQ (4th Generation) at 2.5 GHz).

Accordingly, the nominal capacity of the interfaces of the bundle had been scaled to 100 Mb/s, and we have also scaled the sampling period to 10 s. The occupation of each port of the bundle averaged throughout twelve intervals in ten independent executions is shown in Table 1. Despite the fact that the actual consumption of 100 Mb/s interfaces would be different, this experiment allowed us to validate the behavior of the algorithm.

The results of the real implementation matched the simulation results. We see that GA used three ports to more than 90% of their nominal capacity, one to about 30%, and left the other one unused. These values describe the behavior of a water filling algorithm, as desired per design. BGA avoids having three ports so close to their nominal capacity, although one port still presented an occupation higher than 95%. As the flows were assigned in decreasing rate order, less flows were allocated to the first ports. In fact, in this case, 1.56 were allocated on average to the first port, 6.56 to the second and 96.91 to the third one. The high number of flows allocated to the third port explains why its occupation was so high. CA behaved exactly as desired, using four ports around 80% occupation and leaving the last one empty. The equitable algorithm spread the traffic evenly among all the ports of the bundle. Note that the 0.02% usage of the last port in the three energy-efficient algorithms was due to the flows being assigned randomly during the first interval. The small average occupation differences were mostly due to packet losses, which occurred whenever more than 100 Mb/s were assigned to a port during an interval.

Table 2 collects the average energy consumption averaged throughout the intervals for the same ten independent executions. As we can observe, the differences in the energy consumption among the three energy-efficient algorithms were minimal, and all of them consumed about 18% less than the baseline equitable algorithm. They only differed in the consumption in port 4, which consumed about 7% less with GA than with BGA and CA. This is in accordance to the simulations.

**Figure 16:**
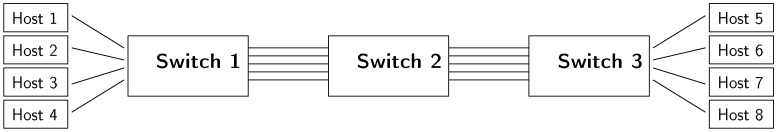
Experimental topology used for QoS-aware algorithms’ validation.

We have also validated the QoS algorithms with the ONOS application using the setup depicted in Figure 16. This time, the setup consisted of three switches (numbered from 1 to 3) and eight hosts (numbered from 1 to 8). Hosts 1 to 4 were connected to Switch 1 and Hosts 5 to 8 are connected to Switch 3. These edge switches were connected to an inner switch by their respective four-link bundles. All the interfaces in this scenario had a nominal capacity of 1 Gb/s.

In this network, three UDP flows without latency requirements were originated in Hosts 1, 2 and 5, with respective destinations in Hosts 5, 6 and 7. These flows have been created with the iperf3 tool. The first two clients send traffic at 700 Mb/s, while the third one at 600 Mb/s. This way we force the flows to be allocated on the first three ports of each bundle. Then, we added three lightweight flows from Host 4 to Host 8 tagged with a predefined differentiated services code point (DSCP)value, so that they can be identified as low-latency by our ONOS application. The purpose of these lightweight flows is to measure the latency suffered by the low-latency packets, using the different scheduling algorithms.

**Figure 17:**
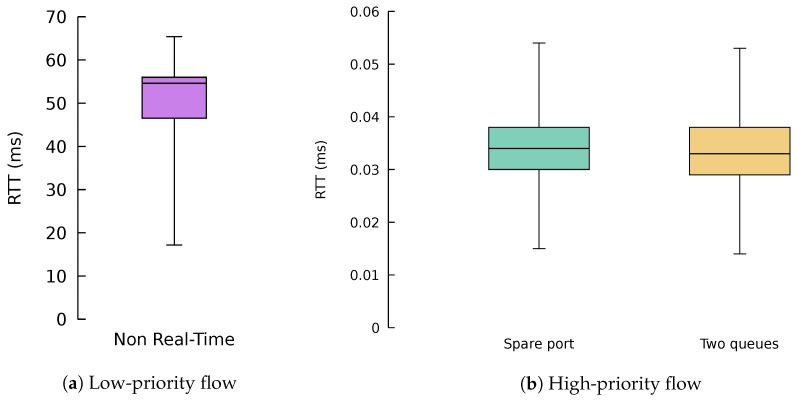
Round-trip time (RTT) for the different algorithms.

Figure 17 shows box and whisker plots with the round-trip time (RTT) of 10,000 packets of the lightweight flows using the different algorithms. The whiskers show that 95% of the samples and outliers have been removed for the sake of clarity. We can see that traffic without real-time requirements suffered a substantial latency in this scenario, around 50 ms. This performance is expected, since the flow was allocated in the same port and queue as the 600 Mb/s big flow. As a result, the packets of the small flows have to contend with the packets of the big flow, yielding considerable waiting times in the queue of the port, which are indeed the main contributions to this large RTT.

Regarding the QoS-aware algorithms, both of them managed to decrease the round-trip time of low-latency traffic by three orders of magnitude in this scenario. The SPA algorithm was using the low-priority queue of the port that does not contain any big flow, thus providing low latency. On the other hand, the TQA algorithm was using the same port as the 600 Mb/s flow, but using the high-priority queue for the lightweight flow rather than the low-priority one as in the case of the big flow. Additionally, despite the algorithms using different ports and queues, their performance in terms of latency was really solid and stable, without major fluctuations, as desired.

### 5.4. Discussion

Simulation results exhibit the existing trade-off between energy consumption, traffic delay and packet losses. Certainly, the analysis shows that CA is the best algorithm. Although the greedy algorithms can be slightly more energy efficient than CA in some scenarios, they could lead to unacceptable packet delays and losses. On the other hand, the computational complexity of the three algorithms is roughly the same. However, CA allows tuning in a fine-grained manner the trade-off between packet delay and energy savings through the safety margin. Increasing the safety margin will contribute to reducing the packet delay and the likelihood of losses, at the cost of a slight increment in the energy consumption in some situations.

It is also important to ponder about the adequate value for the sampling period. Although the use of low values of the sampling period (e.g., 0.01 s) exhibits low delays and packet losses (with the subsequent increase in energy consumption), values lower than 0.5 s are hardly implementable in practice, since they result in a huge (and unmanageable) overhead of control traffic. Moreover, for low sampling periods, this frequent rerouting can harm the performance of TCP, as studied in [30].

## 6. Conclusions

The main focus of this paper has been the minimization of the energy consumption in networking equipment with SDN capabilities when the traffic traverses an aggregate of links between two switches. We elaborated a solution in the form of an SDN application that efficiently concentrates the traffic flows in a few ports of the bundle, dynamically adapting to the variations in the traffic demand. We firstly proposed three allocation algorithms and analyzed them in terms of energy consumption, packet delay and packet losses. We validated the algorithms using real traffic traces through simulation and also in a real implementation on top of the ONOS SDN controller. The obtained results confirm the expected operation of the algorithms, showing that the SDN capabilities of networking equipment can be used to reduce energy consumption in bundles of EEE links up to 50%, without the need for modifying the firmware of the devices.

We also proposed two modifications to the previous algorithms to offer a low-latency service to traffic with stringent QoS requirements while keeping the energy consumption reduced: the SPA algorithm uses the last port of the bundle to transmit high-priority traffic, while the TQA algorithm sets up a low priority and a high priority queue in each output port and transmits low-latency traffic on the high-priority queue. The results showed that the algorithms are able to provide a low-delay service to time-sensitive traffic, achieving a reduction of some orders of magnitude. Moreover, even under the situation of normal traffic congestion, one of the proposals manages to continue offering an accelerated service.

This work could be extended in some lines. First, the centralized view of the whole topology that the SDN controller provides to the applications can be harnessed so that inner switches can reuse the flow allocations performed in the edge switches, in the case where multiple link bundles are present in the network. It will also be interesting to test our solutions in a testbed composed of hardware OF-enabled devices with IEEE 802.3az ports controlled by our ONOS application. Finally, we see a research opportunity in reducing the control plane traffic required, since our work has been focused just on data plane traffic. Minimizing control plane traffic will contribute to the overall energy reduction.

## Figures and Tables

**Table 1:** Average port occupation of the ports of the bundle.

Algorithm	Occupation (%)					
	Port 1	Port 2	Port 3	Port 4	Port 5	Average
Greedy	92.57	97.83	97.05	30.36	0.02	63.57
Bounded-Greedy	83.46	81.16	95.08	61.27	0.02	64.20
Conservative	84.17	83.60	80.78	79.76	0.02	65.67
Equitable	83.89	80.52	54.13	53.63	57.23	65.88

**Table 2:** Average energy consumption of the ports of the bundle.

Algorithm	Energy Consumption (%)					
	Port 1	Port 2	Port 3	Port 4	Port 5	Average
Greedy	99.89	99.99	99.99	92.36	10.24	80.49
Bounded-Greedy	99.80	99.90	99.98	99.38	10.24	81.86
Conservative	99.77	99.92	99.88	99.89	10.24	81.94
Equitable	99.78	99.90	99.04	98.97	99.27	99.39
